# Erastin-induced ferroptosis enhances natural killer cell anti-tumor activity and offers therapeutic potential in neuroblastoma

**DOI:** 10.3389/fimmu.2026.1739503

**Published:** 2026-02-10

**Authors:** Vimala Anthonydhason, Erna Islamagic, Nikki Leijon, Jennie Gaarder, Tommy Martinsson, Susanne Fransson, Fredrik B Thorén, Ganesh Umapathy

**Affiliations:** 1 Centre for Tumor Microenvironment, Barts Cancer Institute, Queen Mary University of London, London, United Kingdom; 2 Tumor Immunology (TIMM) Laboratory at Sahlgrenska Center for Cancer Research, University of Gothenburg, Gothenburg, Sweden; 3 Department of Medical Biochemistry and Cell Biology, Institute of Biomedicine, Sahlgrenska Academy, University of Gothenburg, Gothenburg, Sweden; 4 Department of Laboratory Medicine, Institute of Biomedicine, Sahlgrenska Academy, University of Gothenburg, Gothenburg, Sweden; 5 Department of Clinical Genetics and Genomics, Sahlgrenska University Hospital, Region Vastra Gotaland, Gothenburg, Sweden

**Keywords:** ATF4, ferroptosis, neuroblastoma, NK cell cytotoxicity, ULBP1

## Abstract

High-risk neuroblastoma (NB) often relapses and becomes refractory to treatment, making it a significant contributor to childhood cancer mortality despite its relative rarity. Addressing the challenges posed by NB’s resistance to conventional apoptosis-inducing therapies (e.g., chemotherapy and radiation) has become a pressing concern in pediatric oncology research. In recent years, the growing comprehension of alternative cell death modalities distinct from apoptosis has revealed a promising avenue in the endeavor to combat treatment-resistant cancers. One such mechanism, ferroptosis, has attracted increasing attention for its potential role in combating therapy-resistant cancer cells. High-risk NB typically exhibits an immune-excluded phenotype, characterized by minimal immune cell infiltration. Despite this characteristic, the mechanisms underlying immune exclusion and impaired immune activation in NB remain unclear. Here, we demonstrate that the induction of ferroptosis using Erastin, a cystine-glutamate antiporter inhibitor, reduces NB cell proliferation and foci formation. Furthermore, our transcriptomics analysis revealed that treatment with Erastin in NB cells led to increased expression of ULBP1, a ligand to the activating natural killer (NK) cell receptor, NKG2D. Upon ferroptosis induction, the transcription factor ATF4 was found to drive ULBP1 expression in NB cells. Consistent with this, pre-treatment with Erastin sensitized NB cells to NK cell cytotoxicity in co-culture experiments. These results suggest the NK cell’s cytotoxic function can be enhanced with Erastin-mediated ferroptosis, which may be beneficial for NB patients.

## Introduction

Neuroblastoma (NB), a highly heterogeneous childhood cancer, predominantly affects very young children with a median age of 22 months at diagnosis (1–5). Originating along the sympathetic chain, NB primarily emerges in the adrenal medulla and subsequently disseminates to various regions, including the bone marrow (70%), cortical bone (56%), regional and distant lymph nodes (31%), liver (30%), and intracranial and orbital sites (2–4, 6). Chromosomal aberrations significantly influence NB prognosis. Common alterations associated with poor outcome include deletions of chromosome arms 1p and 11q, gain of 17q, and amplifications of *MYCN* and *ALK* (7–11). Notably, *MYCN* amplification on chromosome 2p24 occurs in approximately 20–30% of NB cases and remains one of the strongest predictors of aggressive disease and poor survival (11–15). In addition to *MYCN*, activating mutations or amplifications of *ALK* contribute to tumor initiation and progression, and together these two alterations often co-occur, defining a particularly high-risk NB subset (7, 8, 11). Such genomic alterations not only drive tumor aggressiveness but also contribute to therapy resistance by deregulating cell death pathways (16, 17).

The immune system also plays a pivotal role in NB progression and treatment response. While infiltration by T cells, dendritic cells, and NK cells is linked to improved prognosis (18, 19), high-risk NB often displays limited immune cell infiltration and reduced MHC class I expression, resulting in an immune-excluded or “cold” tumor phenotype (20, 21). These features contribute to poor responses to immune-based therapies, emphasizing the need to identify strategies that can restore immune recognition and cytotoxic activity. This limitation necessitates the development of innovative therapeutic strategies that enhance apoptosis-inducing agents that modulate the immune system for managing NB patients.

NB, like other solid tumors, requires specific metabolic alterations to fuel its deregulated growth and invasion into surrounding tissues (22, 23). High-risk NB is characterized by an increased production of reactive oxygen species (ROS), which can inflict cellular damage. To counteract this oxidative stress, NB cells undergo metabolic rewiring, relying on cysteine-derived metabolites, including glutathione (GSH), for effective ROS detoxification (22, 23). Most cellular cysteine is acquired through the amino acid transporters, which exchange extracellular oxidized cysteine for intracellular glutamate (24, 25). Erastin, an inhibitor of the cystine-glutamate antiporter (System Xc-), causes depletion of intracellular GSH and has demonstrated several anti-cancer properties, including induction of ferroptotic cell death (26–29).

Ferroptosis is a unique, iron-dependent form of programmed cell death that is driven by lipid peroxidation and distinct from other forms of cell death (26–31). Besides Erastin, ferroptosis can also be induced by inhibiting glutathione peroxidase 4 (GPX4) activity or disrupting GSH synthesis, which ultimately leads to the accumulation of toxic lipid ROS (26–31). Ferroptosis has been increasingly studied as an alternative cancer cell-killing pathway that can overcome the therapeutic insensitivity and resistance of cancer cells to conventional apoptosis-based cancer therapies, prompting its potential use for cancer treatment (26–29). Recent studies have also revealed that ferroptotic cell death can influence the tumor immune microenvironment by releasing stress signals and upregulating immune-activating ligands, thereby bridging metabolic stress with immune recognition (31, 32). However, the mechanisms of ferroptotic cell death and immune modulation remain elusive. In this study, we demonstrate that erastin-induced ferroptosis in NB cells drives upregulation of ULBP1 (UL16-binding protein 1), a ligand for the activating receptor, NKG2D. We further show that Erastin-treated NB cells trigger NK cell cytotoxicity when co-cultured with NK cells.

## Materials and methods

### Cell culture

KELLY and SK-N-BE (2) (*MYCN* amplified), SK-N-AS and SK-N-FI (*MYCN* non-amplified) NB cell lines were employed in this study. All cell lines were cultured in complete media, consisting of RPMI 1640 (Gibco) supplemented with 10% fetal bovine serum (FBS) and 1% penicillin/streptomycin, at 37°C and 5% CO_2_. The natural killer cell line NK-92, generously provided by Prof. Kerry Campbell, was cultured in alpha-MEM containing ribo- and deoxyribonucleosides, supplemented with 12.5% heat inactivated (HI) horse serum (Sigma-Aldrich), 12.5% HI fetal calf serum (FCS), 2.2 g/L sodium bicarbonate, 0.2 mM inositol (Sigma-Aldrich), 2 mM L-glutamine, 0.1 mM 2-mercaptoethanol (Life Technologies), 0.02 mM folic acid (Sigma-Aldrich), and 200 U/mL recombinant IL-2 (Chiron). All cell lines were cultured under standard conditions and routinely tested for the presence of mycoplasma, whereas NB cell line identities were verified by SNP microarray analysis (33).

### Proliferation assay

KELLY, SK-N-AS, SK-N-BE (2), and SK-N-FI NB cell lines were seeded in 96-well plates at a density of 8000 cells/well. The next day, cells were treated with different concentrations of the System Xc inhibitor Erastin (Selleckchem) (range 0.05-10.0 µM). For combination experiments, cells were treated with either Erastin (Selleckchem), the ferroptosis inhibitor Liproxstatin-1 (Selleckchem), or a combination of both. Cellular viability was assessed at 72 h with resazurin assay, with cells incubated with 44 µM resazurin (Sigma Aldrich) for 2-3 h, followed by fluorescence (λex/em=560/590nm) quantification in a TECAN microplate reader.

### Foci formation assay

Cells (1.0 × 10^5^) were seeded in 12-well plates in triplicate and treated with either 1 or 5 µM Erastin with DMSO as a control for 14 days. Plates were washed with PBS and air-dried, then fixed with methanol for 20 minutes. Cells were stained with 0.2% crystal violet in 20% ethanol for 20 minutes, followed by three brief rinses in water. Plates were then scanned using Toshiba Studio 2505AC.

### Western blotting

Proteins from respective cell lines and control cells were extracted using cell lysis buffer (Cell Signaling) supplemented with proteinase and phosphatase inhibitors. Protein amount was quantified using the Pierce BCA Protein assay kit (Thermo Fisher Scientific). The exact amount of total protein sample (20 µg) was separated from each sample using SDS-PAGE and transferred onto a 0.2-µm PVDF membrane. The blots were probed overnight at 4°C with the following antibodies: ULBP1 (#703182, 1:500) from Thermo Fisher, GAPDH (#2118, 1:10,000), and ATF4 (#11815, 1:1,000), which were purchased from Cell Signaling. Horseradish peroxidase–conjugated secondary antibodies, goat anti-mouse immunoglobulin G (IgG) (#sc-2748, 1:5000), and mouse anti-rabbit IgG (#sc-2357, 1:5000) were purchased from Santa Cruz. Protein levels were quantified using ImageJ software.

### RNA interference

ATF4 was silenced in NB cells (KELLY and SK-N-AS) using Silencer Select siRNAs (Invitrogen) according to the manufacturer’s instructions. Cells were seeded in 12-well plates at a density of 3 × 10^5^ cells per well 24 h before transfection, reaching approximately 60-80% confluence at the time of transfection. Transfection was performed using Lipofectamine RNAiMAX (#13778-150, Thermo Fisher Scientific) following the standard protocol. Briefly, siRNA–Lipofectamine complexes were prepared in Opti-MEM (#31985070, Thermo Fisher Scientific) using a final siRNA concentration of 12.5 pmol per well, incubated for 5 min at room temperature, and added dropwise to the cells. A Stealth scrambled siRNA (Invitrogen) was used as a negative control at the same concentration. Cells were incubated for 48 h post-transfection before harvesting for ATF4 and ULBP1 protein expression analysis by immunoblotting.

### Isolation of NK cells

Buffy coats were collected from de-identified healthy donors through the blood center at Sahlgrenska University Hospital. Separation of peripheral blood mononuclear cells (PBMCs) was performed using density gradient centrifugation with Lymphoprep (Stemcell Technologies, #07861). Primary natural killer (pNK) cells were isolated with the Human NK Cell Isolation Kit (Miltenyi Biotec, #130-092-657). The purity of the isolated NK cells was verified by CD56/CD3 staining.

### Cell lysis and degranulation assays with pNK cells

NK cells were pre-stimulated with 500 IU/mL IL-2 for three days prior to use in functional assays. KELLY cells (non-treated and treated with 1 μM Erastin) were used in assays with primary NK cells. KELLY cells were labelled with CellTrace Violet (1:2000, Invitrogen) and co-cultured with NK cells for three hours at the indicated effector-to-target (E:T) ratios. Cells were then stained with Live/Dead-Near IR dye (1:1000, Invitrogen). For degranulation assays, an anti-CD107a antibody (1:100, clone H4A3, BD Biosciences, #565113) was added before co-incubation.

### NK cell cytotoxicity with NK-92 cells

NB cells (KELLY, SK-N-AS, and SK-N-FI) were plated for 48 h in 6-well plates (0.1/0.2x10^6^ cells per well) before 48 h treatment with Erastin (1 μM/1.5 μM) to induce ferroptosis. NB cells were labeled with CellTrace Violet (1:2000, Invitrogen, #C34557), co-cultured with NK-92 cells for three hours at the indicated E:T ratio, and subsequently stained with Live/Dead Near-IR dye (1:1000, Invitrogen, #L34993) and washed twice in PBS to determine the percentage of dead NB cells using flow cytometry.

### Expression of other NKG2D ligands on NB cells and NKG2D receptor on NK-92

SK-N-AS cells were stained for the expression of other NKG2D ligands: ULBP2/5/6 (1:100, clone 16S903, R&D, mouse anti-human, #MAB1298), ULBP3 (1:100, clone 166510, R&D, mouse anti-human, #MAB1517), ULBP4 (1:100, clone 6E6, Santa Cruz Biotechnology, mouse anti-human, #53133), MICA/B (1:20, BD Pharmingen, #558352). NK-92 cells were stained for NKG2D expression (1:20, Miltenyi Biotec, clone REA797, #130-111-645).

SK-N-AS and NK-92 cells were washed twice with PBS and stained with indicated mouse anti-human primary (ULBPs) or directly conjugated antibody (MICA/B, NKG2D) for 30 min at 4°C with L/D Near-IR dye (1:1000, Invitrogen). After two washes with PBS, cells stained with unconjugated primary antibodies incubated with goat anti-mouse secondary antibody (1:500, Invitrogen, A55747) for 30 minutes at 4°C. SK-N-AS cells then washed three times with PBS, and NKG2D ligand and receptor expression was analyzed by flow cytometry.

### Lipid peroxidation detection and ULBP1 expression

To stimulate ferroptosis, KELLY, SK-N-AS, SK-N-BE (2), and SK-N-FI were plated in 6-well plates (0.5x10^6^ cells per well) for 48 h and afterwards treated with indicated Erastin concentrations (KELLY:1.5 µM, SK-N-AS, SK-N-BE (2), and SK-N-FI: 3 µM). For lipid peroxidation measurements, cells (0.1x10^6^ per well) were plated in 96-well plates with the BODYPI™ 581/591 C11 probe (10 µM, Invitrogen, #D3861) and incubated for 5 h at 37°C in the dark prior to repeated PBS washing. By measuring the ratio of green to red fluorescence, it is possible to relatively quantify the extent of lipid peroxidation in live cells or tissue.

Separately, NB cells (KELLY, SK-N-AS, SK-N-BE (2), and SK-N-FI) were seeded and washed with PBS, then stained for 30 minutes at 4°C with anti-ULBP1 primary antibody (1:100, clone 170818, R&D systems, #MAB-1380-500) prior to washing and 30 min incubation with secondary antibody (1:500, Invitrogen, #A55747). After three washing steps with PBS, the MFI of ULBP1-expressing live cells was measured using flow cytometry. Data acquisition was performed using a five-laser BD LSRFortessa (BD Biosciences, NJ, USA), and data analysis was conducted using FlowJo software versions 10.9.0 and 10.10.0.

### RNA-seq analysis

Cell line RNA-Seq: KELLY and SK-N-AS cells were seeded (0.5x10^6^) in 6-well plates in triplicate before treatment with Erastin for 24 hours. Control samples were harvested after 24 h. RNA was extracted using the ReliaPrep RNA Miniprep System (Promega #6111) according to the manufacturer’s protocol. The purified RNA was sent to Novogene UK for library preparation and sequencing. RNA-seq paired-end reads (read length 150 base pairs) were aligned to the human GRCh38 reference genome with an average alignment efficiency of 90.9%. Genes were annotated using GENCODE Release 29/GRCh38.p12 (https://www.gencodegenes.org/human/release_29.html) and quantified with HTSeq (version 2.0.3) (34). Only protein-coding genes were selected for further analysis. Differential gene expression was determined using DESeq2 (version 1.42.1) (35). Differentially expressed genes (DEGs) were identified using a threshold of p-value ≤ 0.05 and fold change ≥ 1.5. Over-representation analysis (ORA) was performed with the clusterProfiler package (version 4.10.1) (36) to identify enriched Kyoto Encyclopedia of Genes and Genomes (KEGG) pathways. Gene Ontology (GO) enrichment analysis was further conducted to predict the biological functions associated with the DEGs. Data visualization, including volcano plots and cnetplots, was carried out using R packages.

### Kaplan-Meier analysis

Kaplan-Meier analysis with calculation of log-rank test for overall and event-free survival probability in relation to the expression levels was performed using the Kaplan-scan cutoff method in ‘R2: Genomic Analysis and Visualization Platform’ (http://r2.amc.nl) with the publicly available datasets ‘Tumor Neuroblastoma-SEQC-498-rpm-Seqnb1’ (n=498) and the Cangelosi - 786 - custom - dgc2102 (n=786). The Kaplan-Scan cutoff method examines every increasing expression value as a cutoff for the log-rank test in order to find the optimal segregation point of two groups based on gene expression. This method then presents the most statistically significant cutoff, along with the corresponding Bonferroni-corrected P-value and the initial non-corrected P-value.

### Statistical analysis

Statistical analyses were performed using either GraphPad Prism 7/8 software or the R statistical package (version 4.0). Statistical tests are indicated in the respective sections and figure captions.

## Results

### High levels of cystine/glutamate antiporter expression correlate with the outcome of NB patients

**Figure 1:**
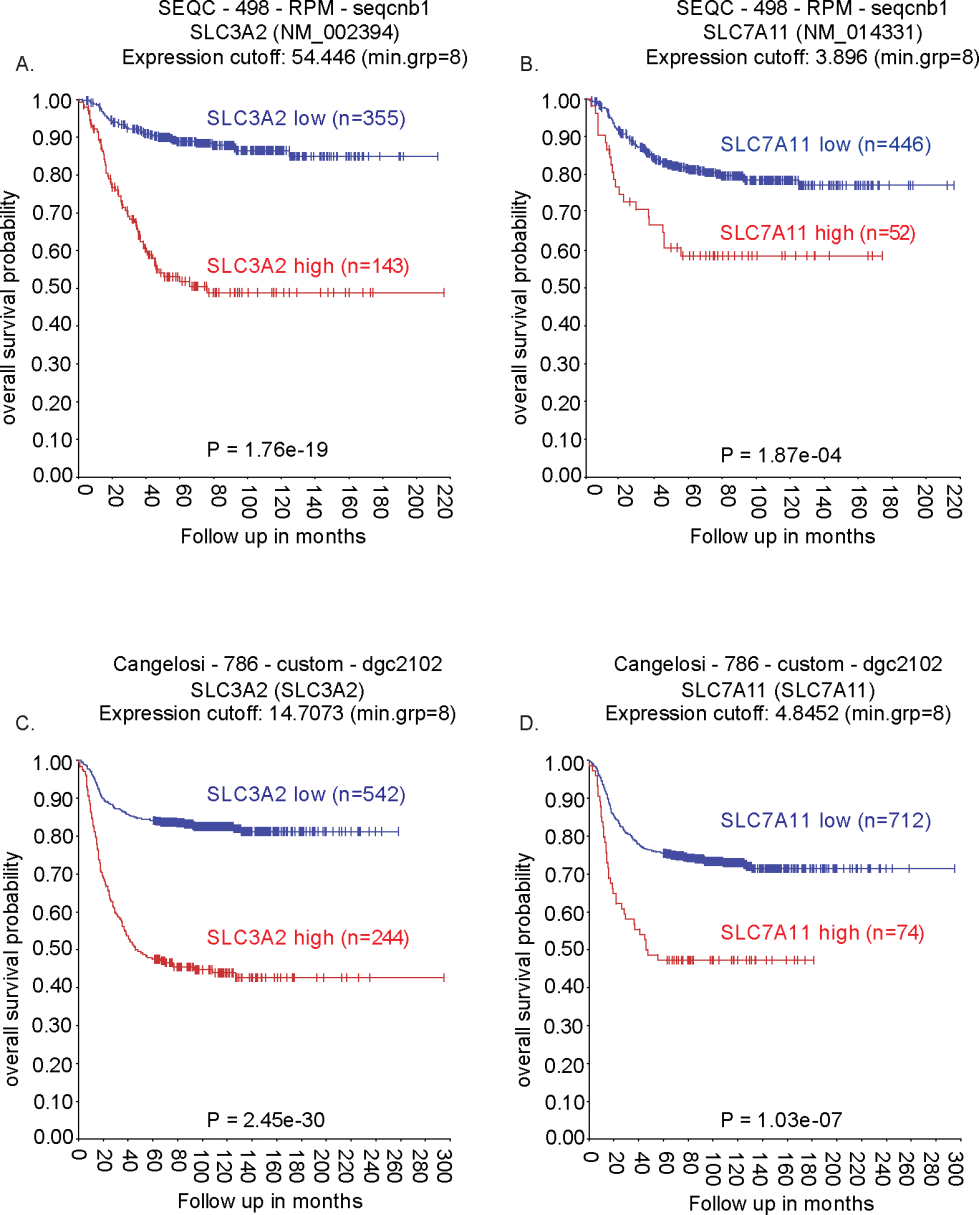
Kaplan–Meier overall survival plots based on SLC3A2 and SLC7A11 expression in neuroblastoma patient cohorts. **(A, B)** Survival analysis of the SEQC cohort stratified by high and low expression of *SLC3A2* **(A)** and *SLC7A11* **(B)** Survival analysis of the Cangelosi cohort stratified by high and low expression of *SLC3A2* **(C)** and *SLC7A11* **(D)**. Sample sizes and P-values are indicated in each panel.

Previous studies have demonstrated that cystine/glutamate antiporter metabolism is a crucial metabolic rewiring process for the progression and survival of NB (22, 23). Taking this into account, we first investigated whether the expression of the cystine/glutamate antiporter genes *SLC3A2 and SLC7A11* correlates with the outcome of NB patients. The relationship between *SLC3A2 and SLC7A11* expression and neuroblastoma survival data was evaluated using the Sequencing Quality Control (SEQC) cohort of 498 patients using the R2 database (http://r2.amc.nl). This revealed a statistically significant correlation between the increased expression of *SLC3A2 and SLC7A11* genes and a poor overall survival (**Figures 1A, B**; P = 1.76e-19 and P = 1.87e-04, respectively). Similar results were obtained from the larger Cangelosi cohort (n=786), which also showed that higher expression levels of both *SLC3A2 and SLC7A11* were significantly associated with poor overall survival rates in NB patients (**Figures 1C, D**; P = 2.45e-30 and P = 1.03e-07, respectively), suggesting that retained glutamate transportation could be associated with poor outcome.

### Erastin induces ferroptosis in NB and reduces cell proliferation

**Figure 2:**
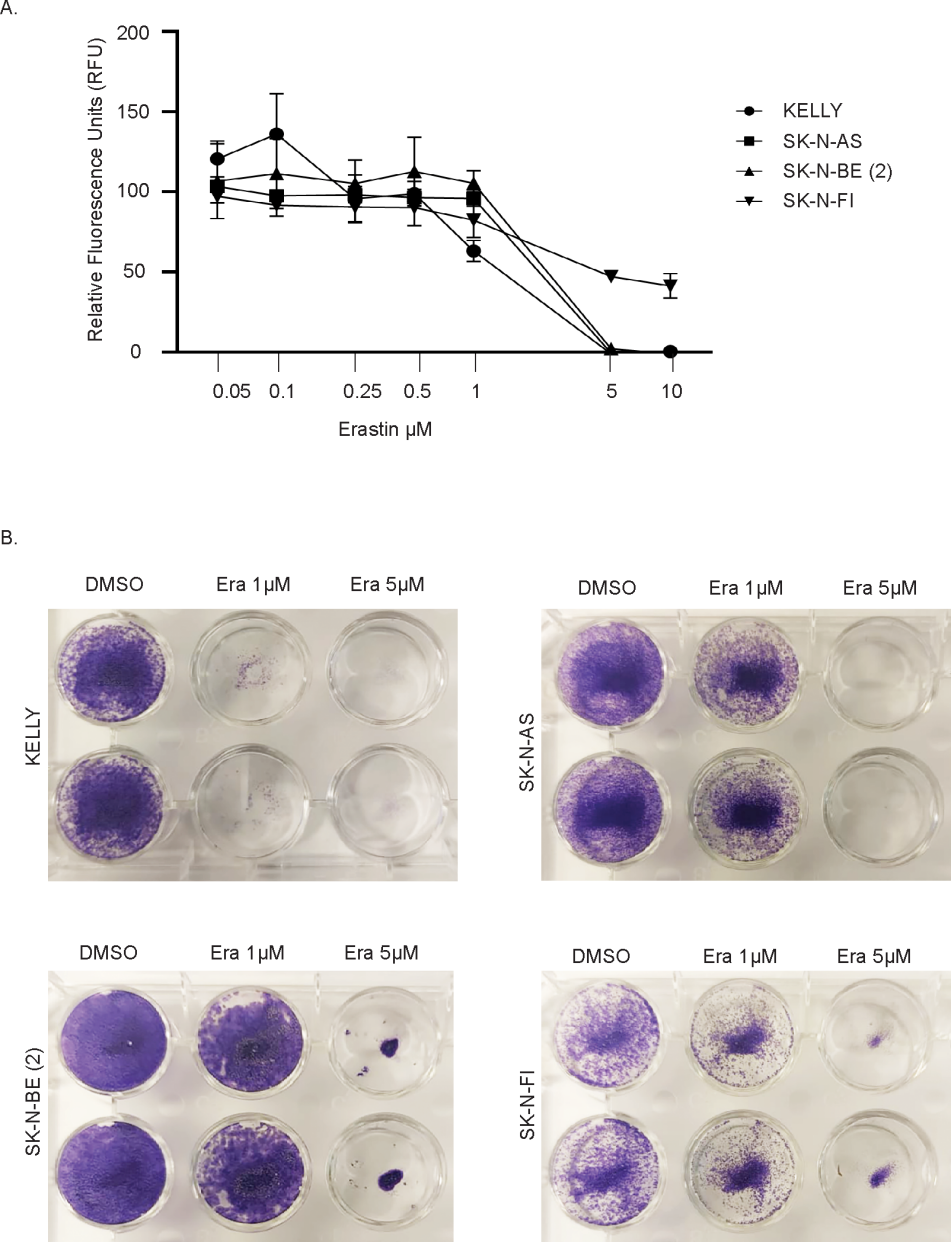
Effect of Erastin treatment on neuroblastoma cell lines. **(A)** Cell viability assay showing relative fluorescence units (RFU) in response to increasing concentrations of Erastin in the indicated NB cell lines. **(B)** Representative images from foci formation assay in the NB cell lines treated with DMSO (control), 1 μM Erastin, or 5 μM Erastin. n = 3 biologically independent experiments.

Next, we set out to investigate whether the cystine/glutamate antiporter inhibitor Erastin affects NB cell growth and foci formation. For this, we used four NB cell lines with different genetic backgrounds: KELLY, SK-N-AS, SK-N-BE (2), and SK-N-FI. Consistent with previous findings (22), Erastin inhibited NB cell growth in a dose-dependent manner. NB cell lines KELLY, SK-N-AS, and SK-N-BE (2) showed similar sensitivity to Erastin. Notably, KELLY cells exhibited a more pronounced decrease in viability even at lower concentrations of Erastin compared to the other NB cell lines, suggesting heightened sensitivity to cystine-glutamate antiporter inhibition. In contrast, SK-N-FI exhibited moderate sensitivity to cystine/glutamate antiporter inhibition (**Figure 2A**). These differences in Erastin sensitivity likely reflect the distinct genetic and metabolic backgrounds of the NB cell lines. We then treated the NB cells with Erastin for a long-term period (14 days), and foci formation was inhibited at both 1 µM and 5 µM concentrations across all four cell lines. Similar to the proliferation assays, KELLY cells showed a marked reduction in foci formation even at lower Erastin concentrations, whereas SK-N-FI cells displayed only moderate sensitivity to Erastin treatment (**Figure 2B**).

**Figure 3:**
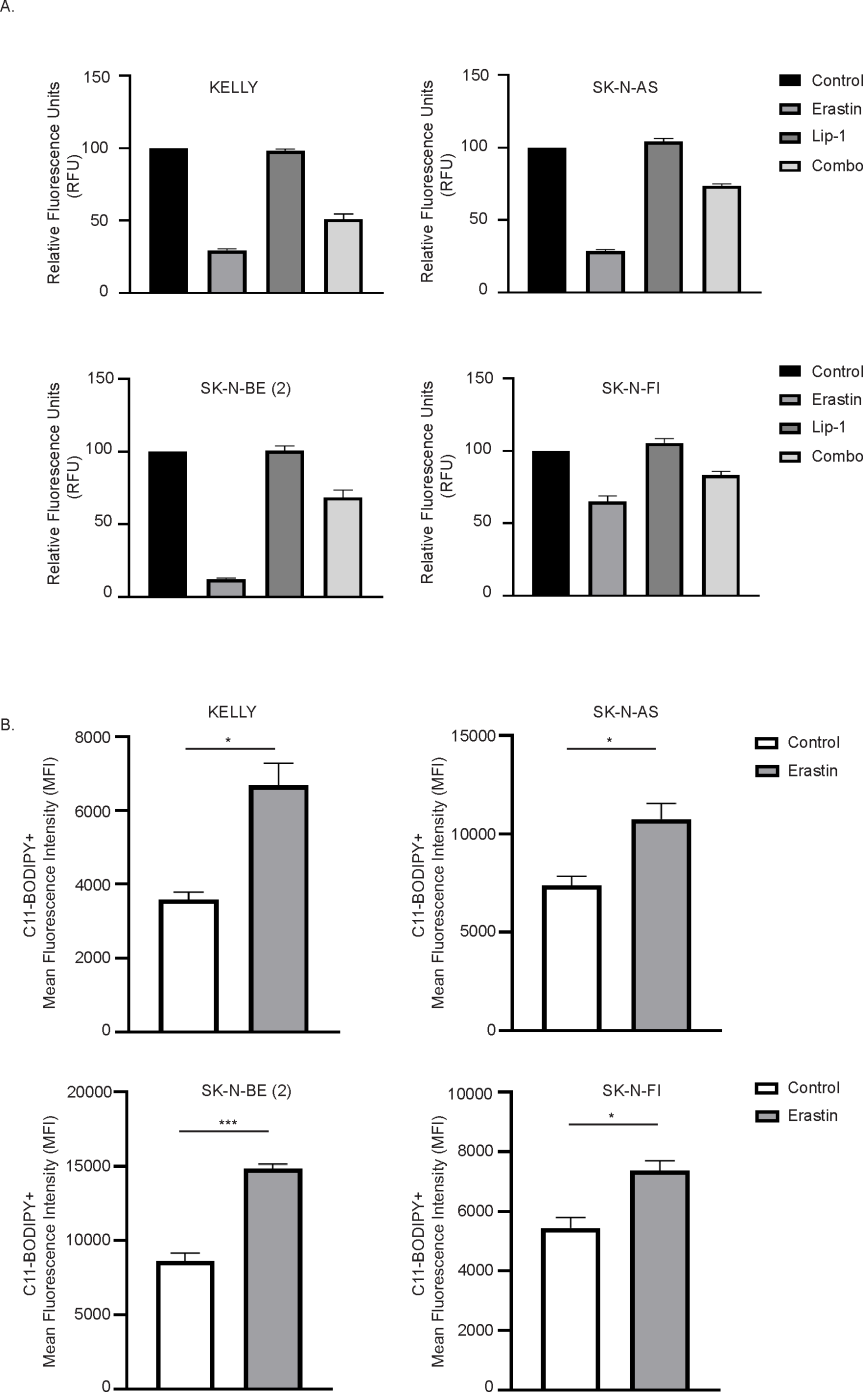
Erastin treatment induces lipid peroxidation in neuroblastoma cells. **(A)** Cell viability of KELLY, SK-N-AS, SK-N-BE (2), and SK-N-FI neuroblastoma cell lines treated with either DMSO (control), Erastin, Liproxstatin-1 (Lip-1), or a combination of Erastin and Lip-1 (1 μM). Viability was assessed using the Resazurin reduction assay. **(B)** Lipid peroxidation levels measured by flow cytometry using the BODIPY™ 581/591 C11 sensor in the indicated NB cell lines after treatment with DMSO or Erastin. n = 3 biologically independent experiments. *P<0.05, ***P<0.001; Paired t test.

Several studies have demonstrated that Erastin, a cystine/glutamate antiporter inhibitor, can induce ferroptosis in both *in vitro* and *in vivo* settings (22, 26, 31). To test this, we treated NB cells with either the ferroptosis inhibitor Liproxstatin-1 (Lip-1), Erastin, or a combination of both, and measured cell proliferation (**Figure 3A**). As seen in **Figure 3A**, the cell death induced by Erastin was prevented when NB cells were treated concomitantly with the ferroptosis inhibitor, Lip-1. The availability of cystine/cysteine is the rate-limiting step in GSH synthesis, which counteracts ROS and prevents ferroptosis — an iron-dependent form of regulated cell death caused by ROS-mediated lipid peroxidation (22, 26, 31). Monitoring ROS formation by flow cytometry using the lipid peroxidation sensor, C11-BODIPY, revealed a significant increase in cellular lipid peroxidation following Erastin treatment (**Figure 3B**). Collectively, these data indicate that the cystine/glutamate antiporter inhibitor Erastin indeed induces ferroptosis in NB cells.

### Transcriptional profiling of ferroptosis induction in NB cells

**Figure 4:**
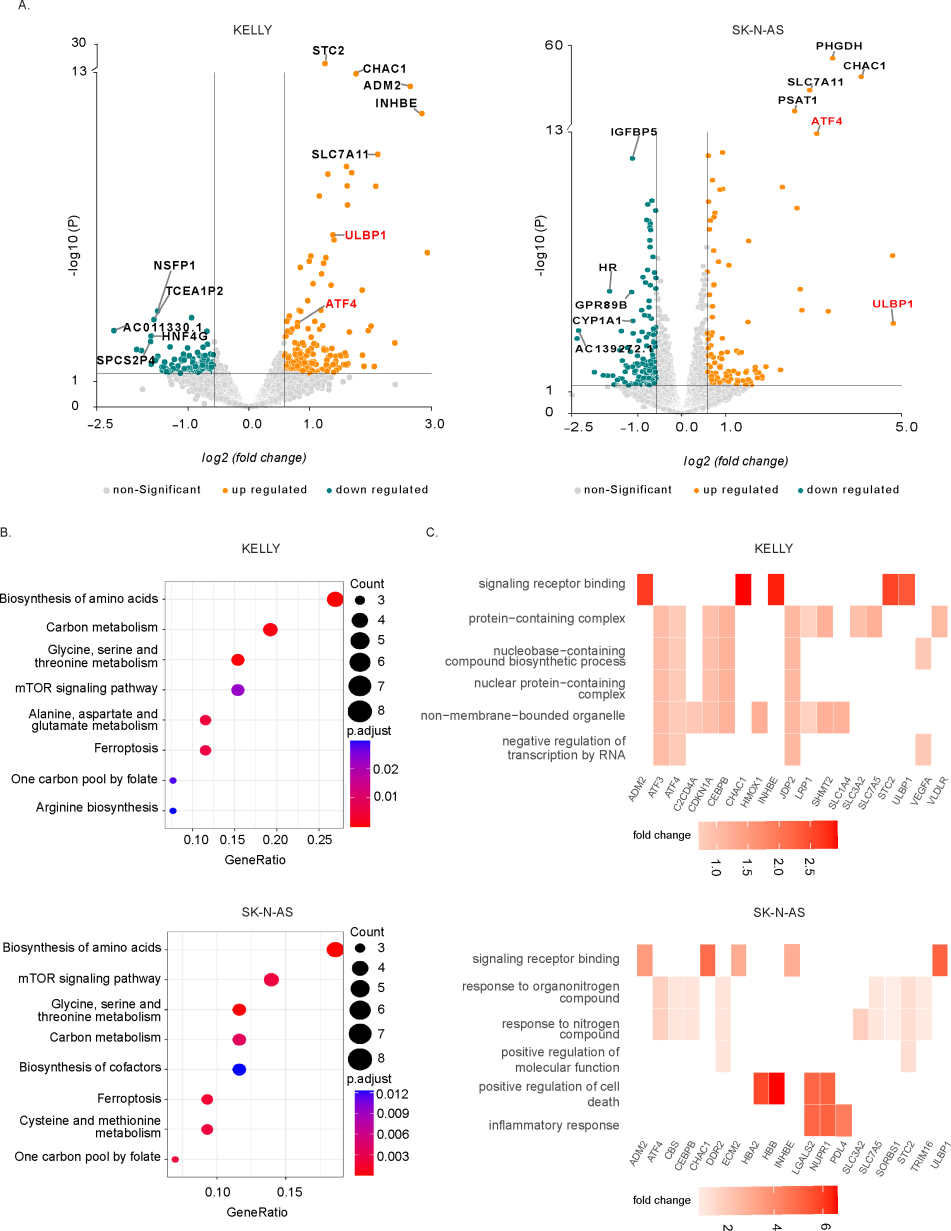
Transcriptomic profiling reveals upregulation of ULBP1 and ferroptosis-related genes following Erastin treatment. **(A)** Volcano plots showing differentially expressed genes in KELLY and SK-N-AS neuroblastoma cell lines treated with 1 µM and 2 µM Erastin for 24 hours, respectively, compared to DMSO control. Non-significant genes are shown in gray; significantly upregulated and downregulated genes are shown in red and blue, respectively. **(B)** KEGG pathway enrichment analysis of DEGs in KELLY and SK-N-AS cells, showing significantly enriched pathways including ferroptosis and amino acid metabolism. **(C)** Gene Ontology (GO) enrichment network highlighting clusters associated with oxidative stress response, amino acid metabolic processes, and signaling receptor binding, with key genes indicated.

To characterize the downstream gene expression response following ferroptosis induction, we performed a comprehensive RNA-Seq analysis after treatment of KELLY (*MYCN* amplified) and SK-N-AS (*MYCN* non-amplified) NB cells with Erastin for 24h (**Figure 4A**). Erastin treatment generated a robust, but variable, response across the two NB cell lines after 24 h. The total number of differentially expressed genes (DEG) (fold change ≥1.5 and P≤ 0.05) in KELLY and SK-N-AS 24 h after treatment with Erastin were 479 and 393, respectively (**Figure 4A**; **Supplementary File 1**).

To elucidate the molecular response following ferroptosis induction, we performed KEGG pathway and Gene Ontology (GO) enrichment analyses on Erastin-treated NB cells. KEGG pathway analysis revealed, not surprisingly, significant enrichment of ferroptosis-associated metabolic pathways, including arginine biosynthesis, one-carbon pool by folate, carbon metabolism, and most notably, the ferroptosis pathway itself (**Figure 4B**). These enriched pathways reflect metabolic reprogramming consistent with oxidative cell death and confirm Erastin’s efficacy in activating ferroptotic signaling in NB cells. Network-based KEGG visualization further highlighted key ferroptosis mediators such as *SLC7A11, SLC3A2*, and *HMOX1*, along with amino acid metabolism genes including *SHMT2, PSAT1*, and *PHGDH*, suggesting a tightly regulated redox stress response (**Supplementary Figures 1A, B**).

To further characterize transcriptional reprogramming, we performed GO term enrichment and network analysis of differentially expressed genes in both KELLY and SK-N-AS cells. The GO network revealed highly interconnected clusters associated with biological processes, including amino acid metabolic processes, and signaling receptor binding (**Figure 4C**). Notably, ATF4, a known transcriptional regulator of the integrated stress response, was prominently enriched within the oxidative stress cluster, supporting its involvement in regulating ferroptosis-associated genes. Among the upregulated genes, *ULBP1* emerged as one of the most prominent hits within the signaling receptor binding category, with robust induction observed in both cell lines following Erastin treatment (**Figures 4A–C**). *ULBP1* encodes a ligand for the NKG2D receptor expressed on NK cells that plays a pivotal role in immune recognition and anti-tumor cytotoxicity, positioning it as a key effector of ferroptosis-linked immunogenic remodeling.

### ULBP1 expression is regulated by ATF4 and linked to poor survival in neuroblastoma

Previous studies have demonstrated that the transcription factor *ATF4* regulates *ULBP1* expression in tumor-derived human cell lines (37, 38). To determine whether this regulatory axis is conserved in NB, we performed siRNA-mediated knockdown of *ATF4* in Erastin-treated KELLY and SK-N-AS cells. Immunoblot analysis revealed that Erastin treatment upregulated both ATF4 and ULBP1 protein levels (**Supplementary Figure 2**), while silencing of *ATF4* markedly reduced ULBP1 protein expression in the presence of Erastin (**Supplementary Figure 2**). These findings indicate that ULBP1 induction in response to ferroptotic stress is ATF4-dependent in NB cells, linking redox-driven stress signaling to immune recognition pathways.

**Figure 5:**
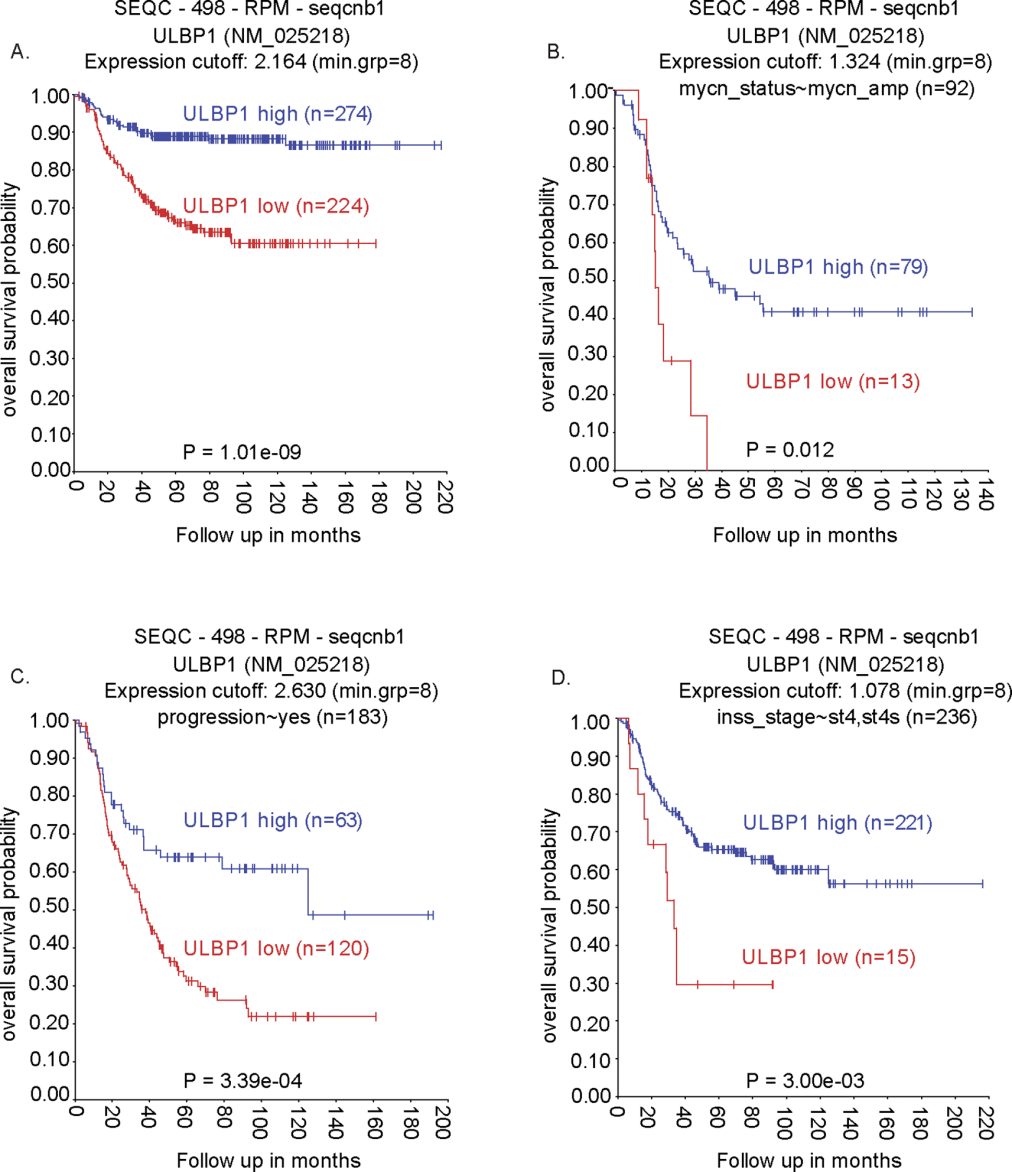
Low ULBP1 expression correlates with poor prognosis in neuroblastoma patients from the SEQC cohort. Kaplan–Meier overall survival analysis was performed using the SEQC neuroblastoma dataset (n = 498) via the R2 Genomics Analysis and Visualization Platform **(A)** Complete cohort; **(B)** *MYCN*-amplified subset; **(C)** Patients with progressive disease; **(D)** INSS stage 4 subset.

To determine the clinical relevance of *ULBP1* expression, we analyzed overall survival data from the Sequencing Quality Control (SEQC) neuroblastoma cohort (n = 498) using the R2: Genomics Analysis and Visualization Platform (http://r2.amc.nl). In the complete cohort (**Figure 5A**), patients with low *ULBP1* expression (n = 224) exhibited significantly poorer overall survival compared to those with high expression (n = 274; P = 1.01e–09). This association remained consistent in clinically aggressive subsets (3, 39) defined by *MYCN* amplification (**Figure 5B**; P = 0.012), with progressive disease (**Figure 5C**; P = 3.39e–04) and, among patients with INSS stage 4 and 4s tumors (**Figure 5D**; P = 3.00e–03). Together, these results highlight *ULBP1* as a potential prognostic biomarker in NB and support its involvement in Erastin-mediated ferroptosis and link to immune activation.

### Validation of ULBP1 in NK cell-mediated neuroblastoma cytotoxicity

ULBP1, a member of the ULBP family, plays a pivotal role in activating natural killer (NK) cells through its interaction with the NKG2D receptor. ULBP1 is a key determinant of tumor immune visibility — while its downregulation supports immune evasion, its expression promotes NK cell-mediated recognition and cytotoxicity, thereby enhancing anti-tumor immune responses. Despite its potential as an immune-modulating factor, ULBP1 expression is frequently reduced in many cancers, thereby impairing NK cell-mediated tumor surveillance. To further understand the expression patterns of NKG2D ligands in NB, we analyzed data from the Human Protein Atlas (40–42). Our analysis revealed that the expression of all NKG2D ligands (including ULBP1-6, MICA, and MICB) was notably lower in NB compared to other cancer types (**Supplementary Figures 3**, **4**).

**Figure 6:**
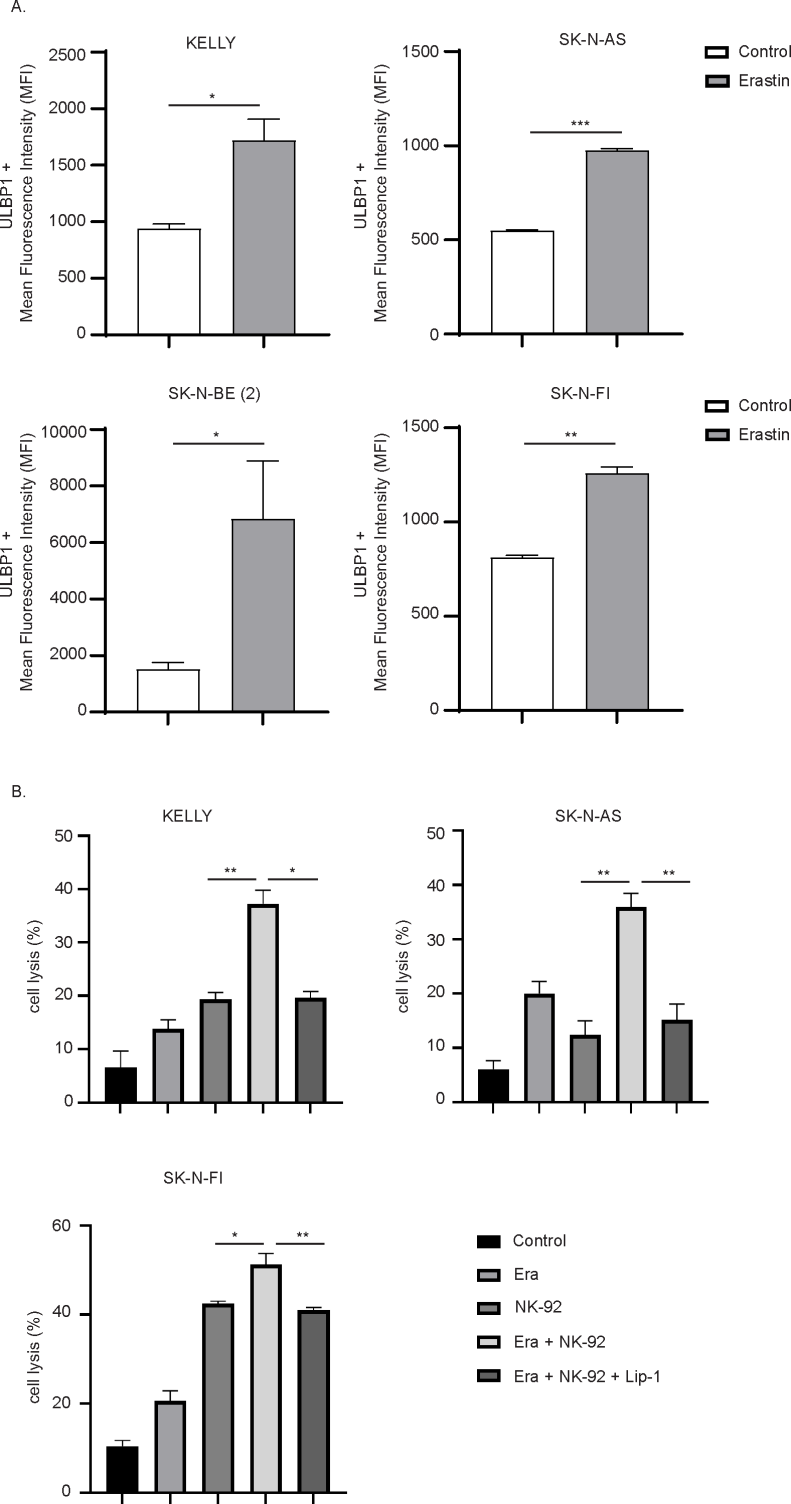
Erastin treatment increases ULBP1 surface expression and enhances NK cell-mediated cytotoxicity in neuroblastoma cells. **(A)** Surface expression of ULBP1 was measured by flow cytometry in KELLY, SK-N-AS, SK-N-BE (2), and SK-N-FI cells treated with DMSO (control) or Erastin. **(B)** NK cell-mediated cytotoxicity assay using NK-92 cells co-cultured with neuroblastoma cells (E:T ratio was 8:1) pre-treated with either DMSO (Control), Erastin, or Erastin in combination with Liproxstatin-1 (Lip-1). Cell lysis percentages were quantified in KELLY, SK-N-AS, and SK-N-FI cells. n = 3 biologically independent experiments. *P<0.05, **P<0.01, ***P<0.001; Paired t test.

To validate the involvement of ULBP1 in ferroptosis-associated immune modulation, its expression was analyzed in four NB cell lines: KELLY, SK-N-AS, SK-N-BE (2), and SK-N-FI. Cells were treated with Erastin, and ULBP1 expression was measured using flow cytometry (**Figure 6A**). Across all four cell lines, a significant increase in ULBP1 expression was observed following Erastin treatment, indicating a consistent upregulation of ULBP1 in cells undergoing ferroptosis (**Figure 6A**; **Supplementary Figure 5A**). To assess whether ferroptotic stress broadly affected NKG2D ligand expression, we additionally examined the surface expression of other NKG2D ligands (ULBP2–6, MICA, and MICB) in SK-N-AS cells following Erastin treatment. Consistent with our RNA-seq data, no significant changes in the expression of these ligands were observed, indicating that Erastin-induced ferroptosis selectively upregulates ULBP1 rather than globally enhancing NKG2D ligand expression (**Supplementary Figure 5B**; **Supplementary File 1**). To explore the potential link between ferroptosis-induced ULBP1 upregulation and NK cell-mediated cytotoxicity, co-culture experiments were performed using NB cells and the NK cell line NK-92. Before co-culture, surface expression of the activating receptor NKG2D on NK-92 cells was confirmed by flow cytometry, verifying their capacity to recognize NKG2D ligand–expressing target cells (**Supplementary Figure 5C**). KELLY, SK-N-AS, and SK-N-FI NB cell lines were pre-treated with Erastin to induce ferroptosis or co-treated with the ferroptosis inhibitor Lip-1. After treatment, NB cells were co-cultured with NK-92 cells, and cytotoxicity was assessed by flow cytometry.

Our results demonstrated that Erastin treatment significantly enhanced NK cell-mediated cytotoxicity in all three NB cell lines (**Figure 6B**). Interestingly, SK-N-FI cells displayed only moderate sensitivity to Erastin in proliferation and foci formation assays, but co-culture with NK cells revealed a significant increase in NK cell-mediated cytotoxicity, indicating that ferroptotic stress can potentiate immune-mediated cytotoxic responses (**Figure 6B**). However, when the ferroptosis inhibitor Lip-1 was added alongside Erastin, the observed enhancement in NK cell cytotoxicity was significantly reduced (**Figure 6B**). To further validate these findings using clinically relevant effector cells, we next performed co-culture experiments with primary NK cells isolated from healthy donors. Consistent with the results obtained using NK-92 cells, Erastin pretreatment of NB cells resulted in a significant increase in primary NK cell–mediated cytotoxicity compared to untreated controls (**Supplementary Figure 5D**). In addition, Erastin pretreatment significantly increased NK cell degranulation, as measured by surface expression of CD107a, indicating enhanced functional activation of primary NK cells upon engagement with ferroptosis-stressed NB cells (**Supplementary Figure 5E**). Collectively, these data demonstrate that Erastin-induced ferroptotic stress enhances NK cell cytotoxic function in both NK-92 cells and primary human NK cells.

## Discussion

This study identifies a novel immune-modulatory mechanism in NB, wherein the induction of ferroptosis by Erastin upregulates ULBP1, thereby enhancing NK cell-mediated cytotoxicity. Our findings add an essential layer to the current understanding of ferroptosis, particularly in pediatric cancers like NB, which are typically resistant to conventional apoptosis-based therapies and exhibit poor immune infiltration profiles (21, 43, 44).

Our results show that Erastin-mediated inhibition of the cystine/glutamate antiporter effectively induces ferroptosis in NB cells, as evidenced by reduced cell proliferation, inhibition of foci formation, and increased lipid peroxidation. These findings align with previous studies reporting ferroptosis induction in glioblastoma and pancreatic cancer; however, our work extends these observations by highlighting a novel immune modulatory role in NB (26, 45). ULBP1 plays a crucial role in tumor immune recognition; however, its expression is often downregulated in cancers as an immune evasion strategy (46–48). In our study, Erastin-induced ferroptosis markedly increased ULBP1 expression at both the transcript and protein levels, suggesting that ferroptotic stress can reactivate silenced immune recognition pathways in NB. This observation gains further relevance when viewed against baseline expression data from the Human Protein Atlas, which shows that NB has notably low expression of NKG2D ligands—including ULBP1–6, MICA, and MICB—compared with most other cancer types. This low baseline aligns with the immune-excluded phenotype commonly observed in NB, characterized by minimal immune infiltration and poor antigen presentation (19, 21, 49). The restoration of ULBP1 expression following ferroptosis induction, therefore, represents a potential mechanism to overcome this inherent immune silence and re-sensitize NB cells to NK cell recognition.

We further identified ATF4 as a key transcriptional mediator of ULBP1 upregulation in the context of ferroptosis. Knockdown experiments confirmed that *ATF4* silencing markedly reduced ULBP1 expression in Erastin-treated cells. This finding is consistent with prior evidence that ATF4 drives the expression of NKG2D ligands under cellular stress (28, 29) and supports the notion that ferroptosis leverages a broader stress-response program to enhance tumor immunogenicity. The prognostic significance of ULBP1 was reinforced by our analysis of the SEQC NB patient cohort, where low ULBP1 expression consistently correlated with poor overall survival across high-risk clinical subsets, including those with *MYCN* amplification and progressive disease. This supports previous findings linking NKG2D ligand loss to tumor aggressiveness and immune evasion in other cancer types (50–52). Importantly, this clinical association likely reflects tumor immunoediting, whereby NB cells with intrinsically high *ULBP1* expression are preferentially eliminated by immune surveillance during early disease, leading to the selective outgrowth of more aggressive, immune-evasive clones characterized by low *ULBP1* expression at advanced stages. Within this framework, our findings suggest that Erastin-induced ferroptosis may therapeutically counteract this acquired immune-evasive state by re-inducing ATF4-dependent ULBP1 expression, thereby restoring tumor cell susceptibility to NK cell-mediated cytotoxicity. Thus, our data indicate that ULBP1 is not only a functional mediator of NK cell activation but also a clinically relevant biomarker for risk stratification of NB.

The co-culture experiments presented in this study provide direct functional evidence that Erastin-induced ferroptosis sensitizes NB cells to NK cell-mediated cytotoxicity. Treatment with Erastin markedly enhanced the susceptibility of NB cells to lysis by NK-92 cells, and this effect was significantly reduced by co-treatment with the ferroptosis inhibitor Liproxstatin-1. Importantly, these findings were further supported using primary NK cells from healthy donors, where Erastin-pretreated NB cells induced a significant increase in NK cell-mediated cytotoxicity accompanied by enhanced degranulation. Notably, both *MYCN* and non-amplified NB cell lines exhibited increased *ULBP1* expression and enhanced NK cell-mediated cytotoxicity upon ferroptosis induction, indicating that this immune-stimulatory effect of Erastin is independent of *MYCN* status. These results confirm that the observed increase in cytotoxicity is specifically dependent on ferroptotic stress. It complements recent findings by Mañas et al., who reported that ferroptosis-inducing drugs can synergize with chemotherapy to overcome treatment resistance in chemotherapy-resistant NB models (53). Together, these studies suggest that ferroptosis inducers could be incorporated into multimodal treatment regimens, not only to enhance cytotoxicity but also to reshape the immune microenvironment.

Furthermore, these experiments validate the immunogenic potential of ferroptosis and underscore the therapeutic promise of exploiting this pathway to improve immune-mediated tumor clearance in NB. Notably, the dual role of Erastin—in inducing ferroptosis and enhancing immune recognition—highlights a therapeutic opportunity to integrate ferroptosis inducers with immune-based treatments. This approach differs from current NB immunotherapy strategies, such as anti-GD2 antibodies or checkpoint inhibitors, which primarily target surface antigens or T-cell responses and have shown limited efficacy in immune-excluded tumors like NB (49, 54, 55). Our findings suggest that combining ferroptosis induction with NK cell-based therapies may synergistically overcome both resistance and immune exclusion. While our data provides mechanistic insight into ferroptosis-induced immune modulation in NB, we acknowledge that the study is limited to *in vitro* cell line models. Validation in *ex vivo* patient-derived tumor cells and *in vivo* models will be critical to establish the physiological relevance of ferroptosis–NK cell crosstalk in high-risk NB, particularly in the context of tumor microenvironmental complexity, immune cell heterogeneity, and potential modulation by soluble NKG2D ligands.

In conclusion, this study uncovers a ferroptosis-driven immune reactivation axis in NB mediated by ATF4-dependent ULBP1 expression. These insights pave the way for exploring ferroptosis inducers as adjuncts to immunotherapies in NB and potentially in other cancers with similar immune-evasive phenotypes. Further validation in *in vivo* models and clinical samples will be essential to translating these findings into therapeutic strategies.

## Data Availability

The datasets generated and analyzed in this study are available in public repositories. The RNA-seq data have been deposited in the NCBI Gene Expression Omnibus (GEO) and are publicly accessible under accession number GSE317656.

## Supplementary material

Supplementary Figure 1 KEGG pathway enrichment of differentially expressed genes in Erastin-treated neuroblastoma cells. **(A)** KELLY and **(B)** SK-N-AS cells treated with 1 μM and 2 μM Erastin for 24 h respectively, showing enriched pathways including ferroptosis, amino acid biosynthesis, one-carbon metabolism, and arginine biosynthesis. Network visualization highlights ferroptosis-related genes (*SLC7A11, SLC3A2, HMOX1*) and amino acid metabolism genes (*PHGDH, PSAT1, SHMT2*) within the enriched pathways.

Supplementary Figure 2 ATF4 knockdown reduces ULBP1 protein expression in Erastin-treated neuroblastoma cells.Western blot analysis of ATF4 and ULBP1 protein levels in KELLY and SK-N-AS cells treated with Erastin following siRNA-mediated silencing of *ATF4*. Cells were transfected with control siRNA (SiC) or two independent *ATF4* siRNAs (Si1 and Si2), then treated with or without Erastin (1 μM for KELLY and 3 μM for SK-N-AS). GAPDH served as a loading control.

Supplementary Figure 3 Comparative expression of ULBP family members across cancer types. Normalized transcript per million (nTPM) values for ULBP1, ULBP2, ULBP3, and ULBP4 were retrieved from the Human Protein Atlas and plotted across a range of malignant and non-malignant tissue types.

Supplementary Figure 4 Expression profile of NKG2D ligands across multiple cancer types. nTPM values for ULBP5, ULBP6, MICA, and MICB were extracted and compared across various tumors.

Supplementary Figure 5 Erastin selectively induces ULBP1 without affecting other NKG2D ligands **(A)** Representative flow cytometry histograms showing surface expression of ULBP1, in NB cell lines under the indicated conditions: untreated and Erastin-treated. **(B)** Representative flow cytometry histograms showing surface expression of NKG2D ligands ULBP2/5/6, ULBP3, ULBP4, and MICA/B in SK-N-AS NB cells under the indicated conditions: unstained control, untreated, Erastin-treated, and Erastin + Liproxstatin-1 (Lip-1). **(C)** NK-92 cells were stained with an anti-NKG2D antibody and analyzed by flow cytometry. Unstained cells were included as a negative control to define background fluorescence. **(D)** Quantification of NK cell–mediated cytotoxicity (E:T ratio was 1:1, n=6) against KELLY NB cells following co-culture with pNK cells (n=6), with or without Erastin pretreatment. **(E)** Flow cytometric assessment of NK cell degranulation (E:T ratio was 1:1, n=6), measured by CD107a surface expression (n=6), following co-culture with Erastin-treated KELLY NB cells. *P<0.05; Paired t test.
