# Supplementary figures and images for: Erastin-induced ferroptosis enhances natural killer cell anti-tumor activity and offers therapeutic potential in neuroblastoma
Source: Front Immunol. 2026 Feb 10;17:1739503. doi: 10.3389/fimmu.2026.1739503 (PMC12929413; doi:10.3389/fimmu.2026.1739503)

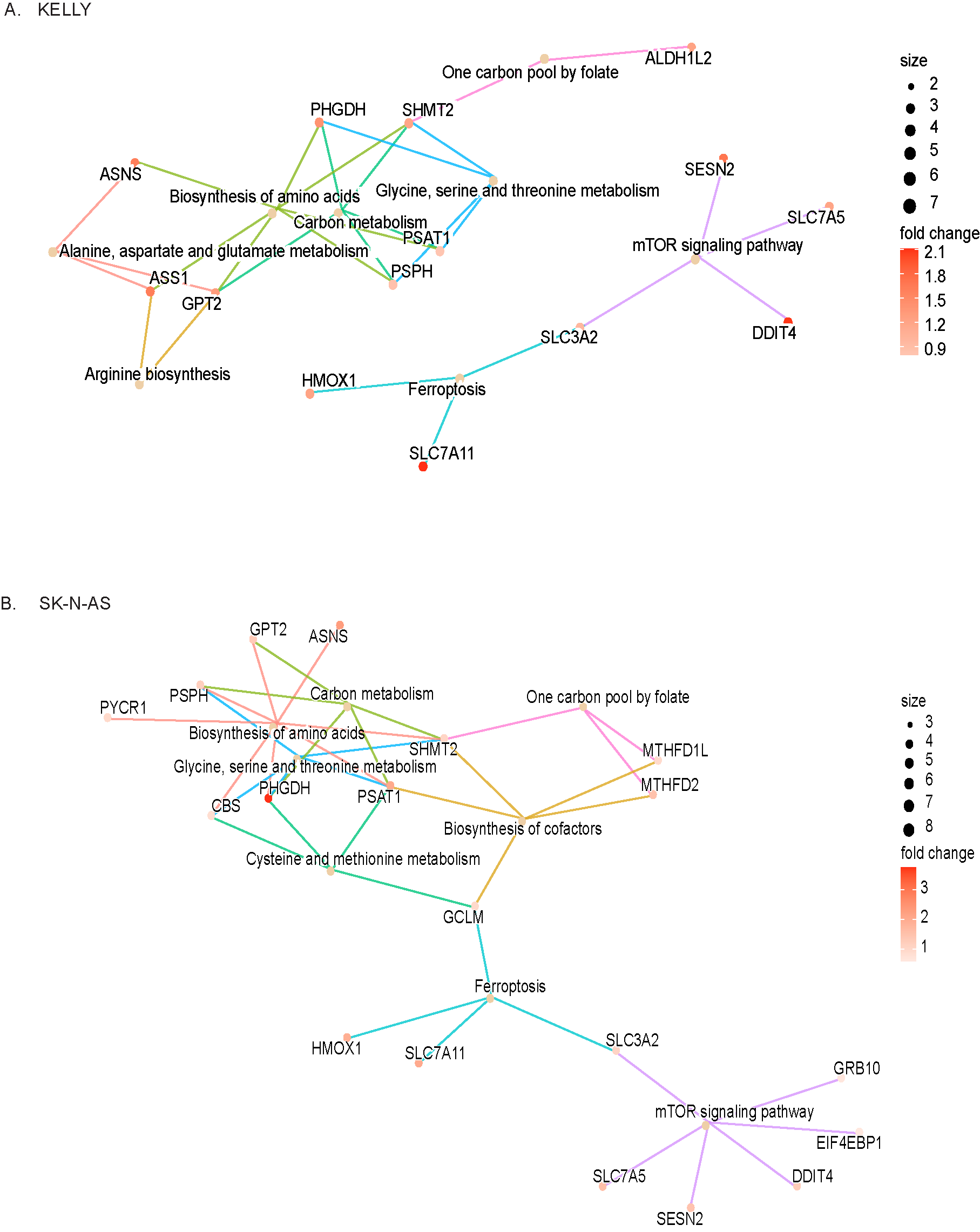

Supplement: Supplementary Figure 1 — KEGG pathway enrichment of differentially expressed genes in Erastin-treated neuroblastoma cells. (A) KELLY and (B) SK-N-AS cells treated with 1 μM and 2 μM Erastin for 24 h respectively, showing enriched pathways including ferroptosis, amino acid biosynthesis, one-carbon metabolism, and arginine biosynthesis. Network visualization highlights ferroptosis-related genes (SLC7A11, SLC3A2, HMOX1) and amino acid metabolism genes (PHGDH, PSAT1, SHMT2) within the enriched pathways. [file Image1.tif]

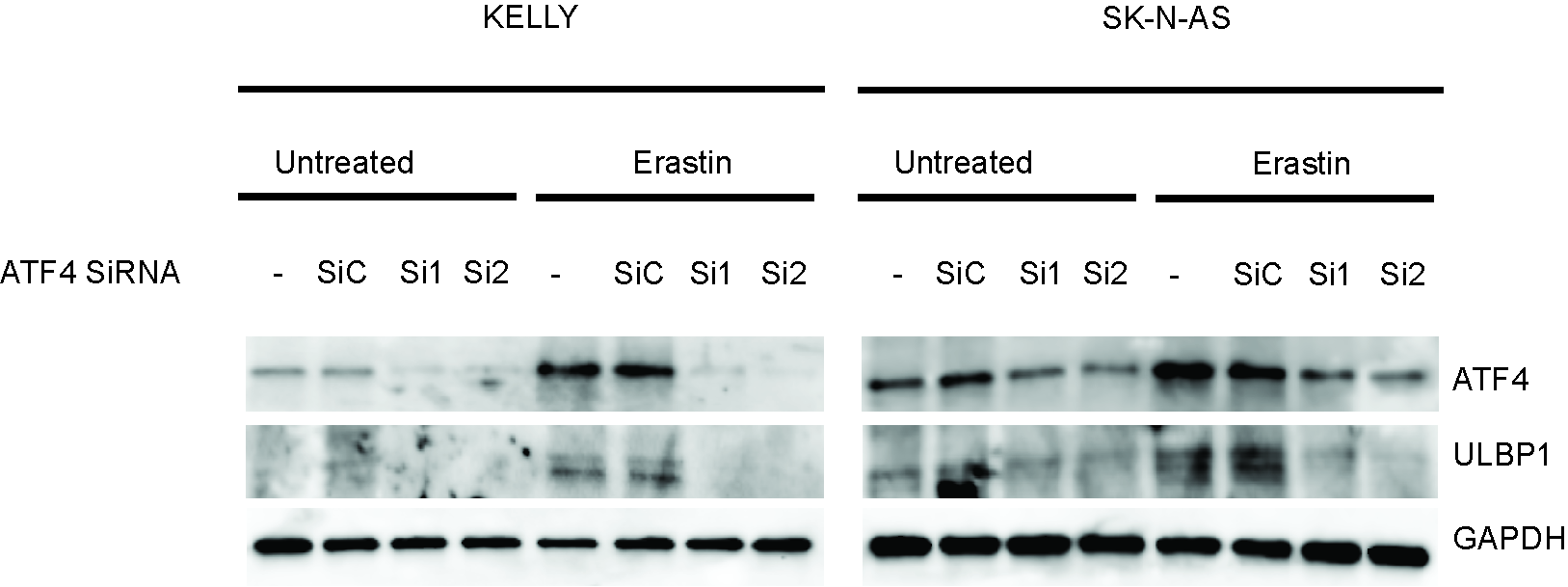

Supplement: Supplementary Figure 2 — ATF4 knockdown reduces ULBP1 protein expression in Erastin-treated neuroblastoma cells.Western blot analysis of ATF4 and ULBP1 protein levels in KELLY and SK-N-AS cells treated with Erastin following siRNA-mediated silencing of ATF4. Cells were transfected with control siRNA (SiC) or two independent ATF4 siRNAs (Si1 and Si2), then treated with or without Erastin (1 μM for KELLY and 3 μM for SK-N-AS). GAPDH served as a loading control. [file Image2.tif]

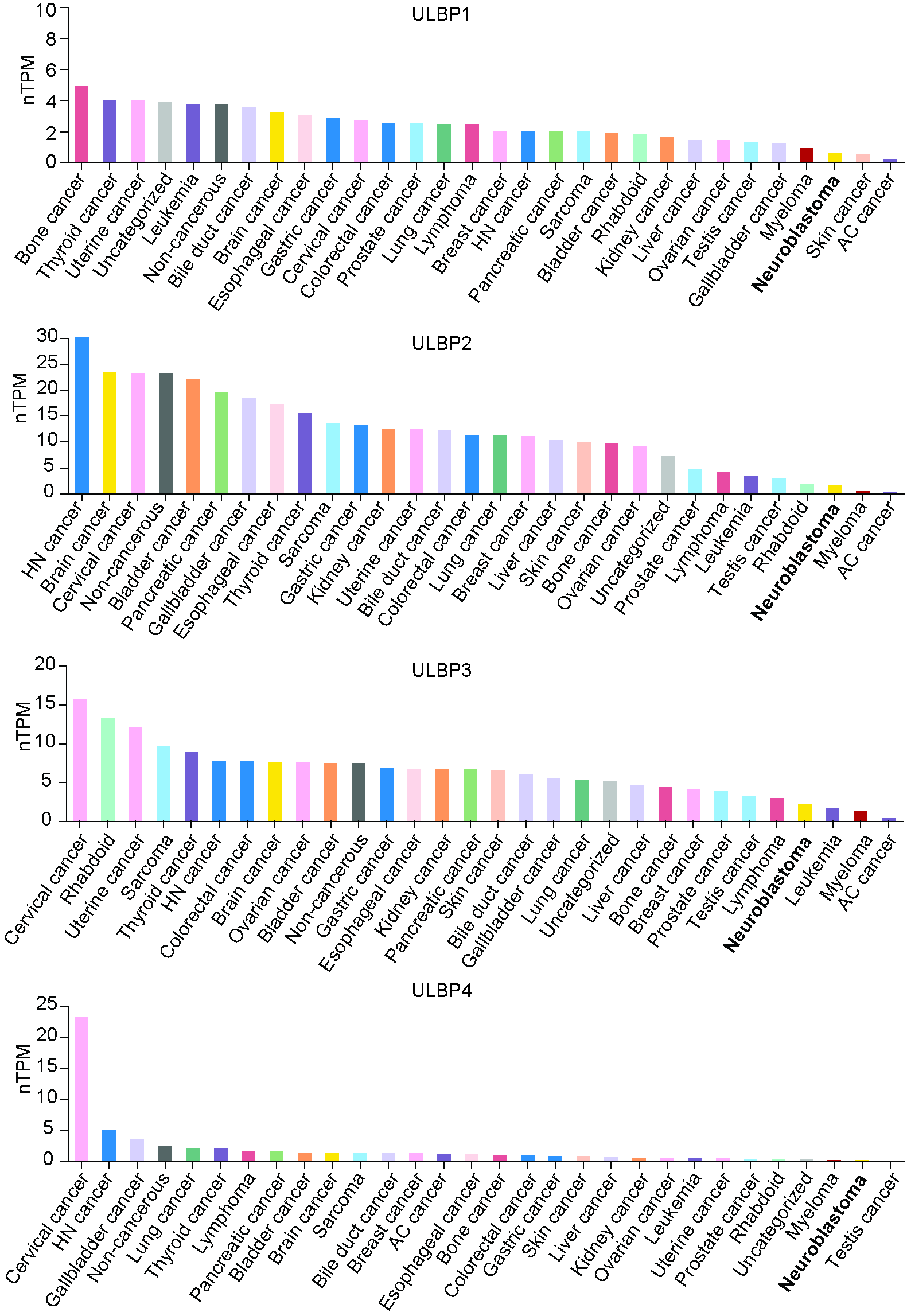

Supplement: Supplementary Figure 3 — Comparative expression of ULBP family members across cancer types. Normalized transcript per million (nTPM) values for ULBP1, ULBP2, ULBP3, and ULBP4 were retrieved from the Human Protein Atlas and plotted across a range of malignant and non-malignant tissue types. [file Image3.tif]

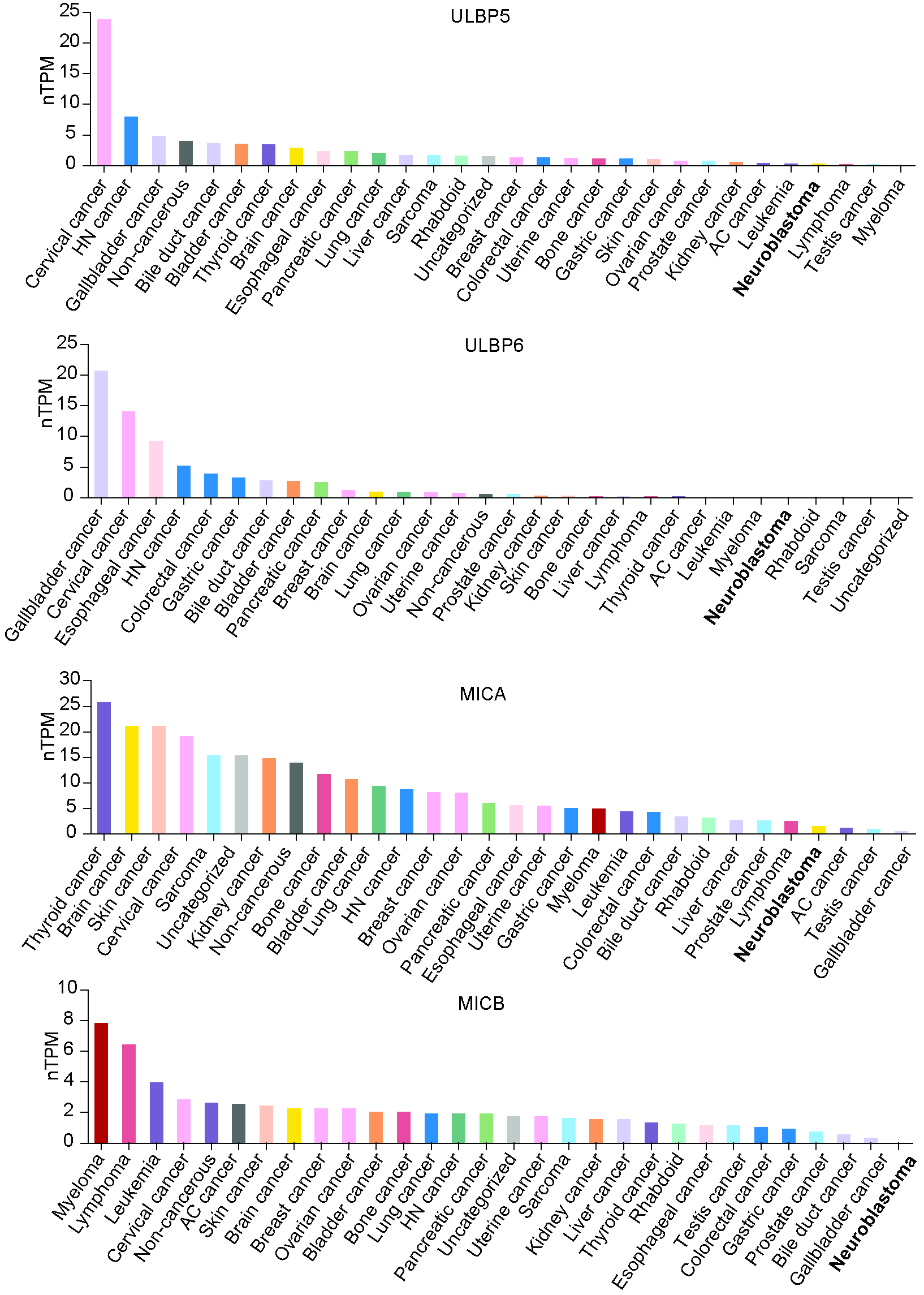

Supplement: Supplementary Figure 4 — Expression profile of NKG2D ligands across multiple cancer types. nTPM values for ULBP5, ULBP6, MICA, and MICB were extracted and compared across various tumors. [file Image4.tif]

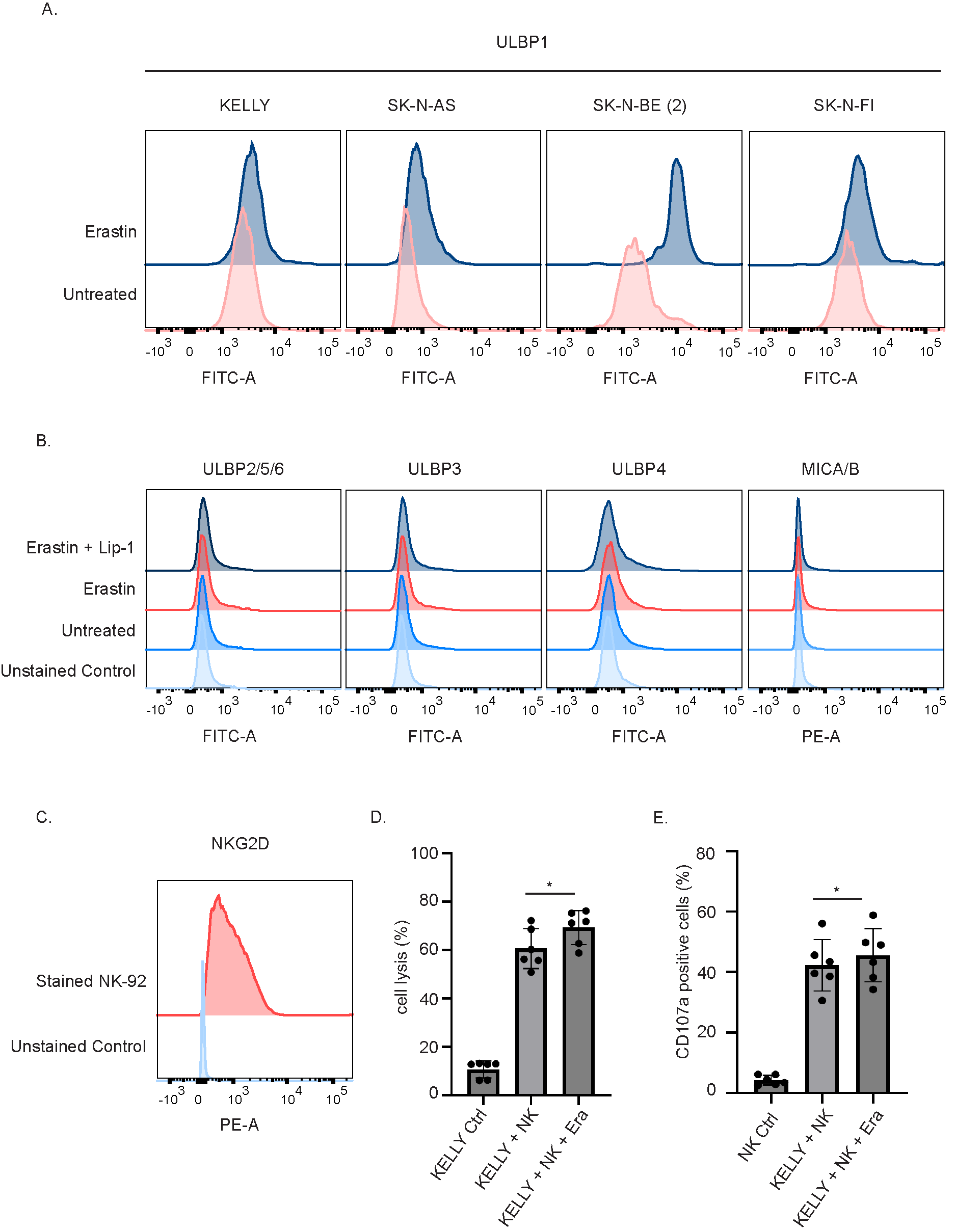

Supplement: Supplementary Figure 5 — Erastin selectively induces ULBP1 without affecting other NKG2D ligands (A) Representative flow cytometry histograms showing surface expression of ULBP1, in NB cell lines under the indicated conditions: untreated and Erastin-treated. (B) Representative flow cytometry histograms showing surface expression of NKG2D ligands ULBP2/5/6, ULBP3, ULBP4, and MICA/B in SK-N-AS NB cells under the indicated conditions: unstained control, untreated, Erastin-treated, and Erastin + Liproxstatin-1 (Lip-1). (C) NK-92 cells were stained with an anti-NKG2D antibody and analyzed by flow cytometry. Unstained cells were included as a negative control to define background fluorescence. (D) Quantification of NK cell–mediated cytotoxicity (E:T ratio was 1:1, n=6) against KELLY NB cells following co-culture with pNK cells (n=6), with or without Erastin pretreatment. (E) Flow cytometric assessment of NK cell degranulation (E:T ratio was 1:1, n=6), measured by CD107a surface expression (n=6), following co-culture with Erastin-treated KELLY NB cells. *P<0.05; Paired t test. [file Image5.tif]
